# Potentials of Neuropeptides as Therapeutic Agents for Neurological Diseases

**DOI:** 10.3390/biomedicines10020343

**Published:** 2022-02-01

**Authors:** Xin Yi Yeo, Grace Cunliffe, Roger C. Ho, Su Seong Lee, Sangyong Jung

**Affiliations:** 1 Institute of Molecular and Cell Biology (IMCB), Agency for Science, Technology and Research (A*STAR), Singapore 138667, Singapore; xinyi.yeo12@sps.nus.edu.sg (X.Y.Y.); grace.cunliffe@postgrad.manchester.ac.uk (G.C.); 2 Department of Psychological Medicine, Yong Loo Lin School of Medicine, National University of Singapore, Singapore 119228, Singapore; pcmrhcm@nus.edu.sg; 3 Division of Neuroscience and Experimental Psychology, School of Biological Sciences, Faculty of Biology, Medicine and Health, University of Manchester, Manchester M13 9PL, UK; 4 Institute for Health Innovation & Technology (iHealthtech), National University of Singapore, Singapore 117599, Singapore; 5 NanoBio Lab, Institute of Materials Research and Engineering (IMRE), Agency for Science, Technology and Research (A*STAR), Singapore 138667, Singapore; 6 Department of Physiology, Yong Loo Lin School of Medicine, National University of Singapore, Singapore 117593, Singapore

**Keywords:** neuropeptide, neuromodulation, neurological disorder, neurotherapeutics

## Abstract

Despite recent leaps in modern medicine, progress in the treatment of neurological diseases remains slow. The near impermeable blood-brain barrier (BBB) that prevents the entry of therapeutics into the brain, and the complexity of neurological processes, limits the specificity of potential therapeutics. Moreover, a lack of etiological understanding and the irreversible nature of neurological conditions have resulted in low tolerability and high failure rates towards existing small molecule-based treatments. Neuropeptides, which are small proteinaceous molecules produced by the body, either in the nervous system or the peripheral organs, modulate neurological function. Although peptide-based therapeutics originated from the treatment of metabolic diseases in the 1920s, the adoption and development of peptide drugs for neurological conditions are relatively recent. In this review, we examine the natural roles of neuropeptides in the modulation of neurological function and the development of neurological disorders. Furthermore, we highlight the potential of these proteinaceous molecules in filling gaps in current therapeutics.

## 1. Introduction

The accelerated acquisition of scientific knowledge and development of modern medicine have resulted in numerous miracles over the past 20 years. For example, new treatment interventions have reduced mortality due to cardiovascular diseases by 40% [1], and targeting specific molecular pathways with small molecule drugs or biologics has turned some cancers from a death sentence into a chronic condition [2,3]. Furthermore, the breakthrough in mRNA vaccine technology has enabled the global COVID-19 pandemic situation to become more manageable [4]. However, there has been a comparative lack of progress in treatment methods and cures for neurological conditions and an exceptionally high failure rate of late-stage clinical trials for neurological and psychiatric diseases [5]. The complexity of neurological processes, irreversible nature of neurological decline [6,7], and presence of a highly selective blood-brain barrier (BBB) [8] have rendered conventional small molecule drugs woefully inadequate in resolving the root aetiology of neurological conditions, often resulting in unwanted neurological impact. It is expectable that drugs exhibiting demonstrable effects in palliative treatments offer no significant improvements for neurological diseases.

Small peptide drugs provide an alternate avenue for the development of novel therapeutics targeting neurological conditions. As intrinsic signalling molecules in normal cellular function, natural peptides or their mimetics mirror physiological modulation of organ or cellular processes [9] and provide specificity unachievable by small molecule drugs developed or repurposed for a specified function. Furthermore, the short, defined sequences of neuropeptides can be used or modified directly during drug synthesis, reducing the need for further structural optimization for functionality. Methods for peptide therapeutics originated from the synthesis of insulin and adrenocorticotropic hormone (ACTH) for treating type I diabetes and endocrine disorders in human patients during the 1980s and 1990s [10]. There are now over 80 peptide drugs available on the market, with many more undergoing development in clinical and preclinical trials [11]. Of these drugs, 65% were approved from the early 21st century, reflective of the growing peptide drug market. A summary of the current U.S. Food and Drug Administration (FDA) approved peptide-based therapeutics is presented in Table 1. The full list of peptide and protein therapeutics approved by the FDA thus far can be found in the THPdb database (http://crdd.osdd.net/raghava/thpdb/, last accessed on 29 November 2021 [12]).

Neuropeptides are small endogenous protein messengers synthesized and secreted by neurons through the regulated secretory route [37]. They are produced widely in the nervous system as pro-peptides, which are further cleaved into smaller fragments, commonly between 5 and 80 amino acids, through a multistep process. They are then matured via post-translational modifications [37]. Multiple peptide products can be generated from a single, larger pro-peptide, and each of these products can function as an independent neuropeptide. For example, opioids acting on classical opioid receptors are produced from one of the three pro-peptides proenkephalin, prodynorphin, or proopiomelanocortin [38]. Mature peptides exert their effects through direct autocrine signalling or indirect paracrine effects on neurons, astrocytes, and microglia nearby. Specifically, peptides that affect neuronal function, but are not produced by neurons, are not considered neuropeptides.

The differential storage and release of neuropeptides compared to neurotransmitters has been reviewed by Merighi et al. (2011) [39]. As opposed to the storage of neurotransmitters in small secretory vesicles, neuropeptides are stored in large granular vesicles (LGVs). Due to the relatively larger size of neuropeptides, their exocytosis is not likely to occur via the ‘kiss and run’ vesicle dynamics reported for neurotransmitter release and instead probably requires complete fusion of the LGV with the pre-synaptic membrane [40]. Neuropeptides can coexist with neurotransmitters in the same neuron, yet their storage in separate vesicles enables selective release, dependent on intrinsic cellular mechanisms and differential calcium release [39]. When combined, the release of neuropeptides and neurotransmitters enables both fast (milliseconds) and prolonged (seconds to minutes) modulation of brain circuits. Differences in neuropeptides and neurotransmitters indicate that both their independent function and interaction are essential for normal brain functioning. The neuropeptide family comprises a broad range of proteinaceous molecules with known pleiotropic effects in development, reproduction, physiology, and behaviour [41,42,43]. Specifically, they can act as hormones, neurotransmitters, and neuromodulators in the nervous system. Hence, they are good candidates for novel drug development for various neurological conditions. The involvement of druggable G protein-coupled receptors (GPCR) in neuropeptide signalling [44] and the small size of neuropeptides further support the suitability for direct use of native neuropeptide sequences in drug development.

In this review, we will examine the role of neuropeptides in neurological development, function, and disease. With a renewed knowledge of neuropeptides in neurophysiology, we are interested in understanding how specific neuropeptides can be employed to combat neurological disorders and how recent advancements in technology and methods of peptide synthesis may push the development of neurological-friendly peptide therapeutics towards a reality. Due to the vast amount of research available relating to neuropeptide function, it would be almost impossible to review a topic so broad to an all-encompassing extent. Alternatively, this review aims to provide a more general outlook of the diversity of the vibrant neuropeptide field. For more detailed discussions of the topics presented, we encourage readers to refer to review papers pointed to in the text.

## 2. Function of Neuropeptides in the Neurological System

Neurological research and the development of neurological therapeutics are dependent on the knowledge of chemical neurotransmission. The awarding of the Nobel Prize in 1936 to Sir Henry Dale and Otto Loewi for their work on acetylcholine’s role in parasympathetic nervous system neurotransmission challenged the initial notion that neurons communicate through direct electrical transmission and set the foundation for the construction of a chemical-based novel for neurological function [45]. Early studies that show the correlation between animal behaviour [46,47] and the levels of brain chemicals, alongside observations that specific classes of drugs mimic the effects of chemical neurotransmission systems, further supported this proposition [48]. The development of methods to detect chemical release from neuronal axon terminals and evidence from neurophysiological measurements eventually confirmed the role of neurotransmitters in neurological function. The story concluded with observations by Arvid Carlsson, Paul Greengard, and Eric Kandel that specific neurotransmitter systems drive critical aspects of animal physiology, behaviour, and cognition. 

It is now well-recognized that the neurotransmitter system is fundamental for normal nervous system development and function. A significant number of drugs developed for treating neurological conditions work based on classical neurotransmitter modulation. For example, galantamine, a reversible cholinesterase inhibitor manufactured under the trade name of Reminyl [49], is prescribed for Alzheimer’s disease (AD) management, and fluoxetine, a selective serotonin reuptake inhibitor also known as Prozac [50], is prescribed to teens suffering from depression. However, neurotransmitters are not the only chemical signalling molecules used by the nervous system. Larger peptide molecules with a longer life span and a larger area of influence are released concurrently with small molecule neurotransmitters [37]. 

### 2.1. Neuropeptidergic Modulation of Neurodevelopment

Brain development occurs soon after conception and persists throughout the lifetime of an organism. The neurodevelopmental process involves dynamic neuronal production, migration, and communication precisely orchestrated by the timed expression or effects of chemical factors in the neurological system. Vasoactive intestinal peptide (VIP), a neuropeptide often associated with gastrointestinal and circadian regulation, guides neuronal differentiation and glia-dependent neuronal survival during neural tube closure in early embryogenesis [51]. The inhibition of VIP activities during gestation results in premature neuronal differentiation, which leads to microcephaly [52] and various behavioural deficits reminiscent of Down syndrome [53]. Furthermore, increased VIP and VIP receptor 2 (VPAC2) levels in newborns are associated with an increased chance of developing autism spectrum disorder (ASD) [54,55,56], further supporting the role of VIP in neurodevelopment. Although initially supplied by the maternal uterine tissue [57], VIP is produced by the central nervous system to regulate neuronal and synaptic activity later in development. For example, it is implicated in the development and control of circadian rhythms and is expressed by neurons in the suprachiasmatic nucleus of the hypothalamus alongside gastrin-releasing peptide (GRP) and arginine vasopressin (AVP) [58]. VIP-expressing neurons have been reported to be important for the maturation of SCN neural networks during development [59]; one study has shown that the ablation of VIP neurons dramatically altered circadian gene expression in neonatal mice [60]. 

Although not essential for early nervous system development, peptide hormones such as oxytocin and vasopressin are involved in the experience-dependent maturation of the neuronal circuit. Oxytocin has time-specific effects on early postnatal development associated with multimodal sensory processing and integration [61]. This is likely due to the combinatorial effect of various neuronal signalling components and neural substrates involved in the stress response, such as the oestrogen receptor, vasopressin system, cortisone, and ACTH [62]. Similar to VIP, polymorphisms in the OXTR gene encoding the oxytocin receptor have been linked to the development of ASD [61,63,64,65], a further reflection of the neuropeptide’s role in neurodevelopmental processes. However, it is unclear why the modulation of oxytocin levels may lead to such alterations in neurodevelopment associated with social behaviours. Several studies (compiled and reviewed by Rajamani et al.) [66] have shown that oxytocin alterations impact both long-term potentiation (LTP) and long-term depression (LTD) of synapses during early development. Additional neuropeptides which have been suggested to play a role in neuronal synaptic plasticity development include neuropeptide Y (NPY), pituitary adenylate cyclase-activating polypeptide (PACAP), and TLQP-62 in complementary pathways contributing to early hippocampal neurogenesis [67], neuron differentiation, and neurite outgrowth during development [68,69].

Following the cessation of fetal and early postnatal development, the adult nervous system retains neurogenic potentials in restricted brain regions. The dentate gyrus contains a rich reservoir of NPY-producing gamma-aminobutyric acid (GABA)-ergic interneurons [70]. NPY generation is sensitive towards changes in hippocampal neuronal activity [71] and associated with a robust enhancement in granule cell neurogenesis [72], likely resulting from an increase in ERK1/2-dependent proliferation of neural stem cells [73]. Interestingly, a comparison between various neurogenic populations of the adult brain revealed the involvement of the NPY receptor Y1 (Y1R) in neuronal precursor cell proliferation and differentiation [74,75]. Although NPY or Y1R-deficient mice develop significantly lower numbers of olfactory neuron precursors [74] and present with an absence of NPY-induced dentate gyrus cellular proliferation [73], there are no overt changes in memory acquisition [76]—a phenomenon linked to enhancements in neurogenesis [77]. It is not clear if the preferential recruitment of Y1Rs during neurogenesis is due to a predominant recruitment of neural stem cells expressing the Y1R since Y1, Y2, and Y5 receptors are found throughout the nervous system [78]. 

### 2.2. Involvement of Neuropeptides in Normal Neurological Homeostasis

The contribution of neuropeptides to neurological function does not end with the conclusion of development. Neuropeptides further regulate neuronal processes to prevent the runaway of neurological activity and behavioural dysregulation. They contribute to homeostatic processes controlling ion channel activity and expression, synaptic scaling, inhibitory synaptic activity, and neurotransmitter release factors fundamental to neuronal activity and function [79]. The plethora of neuropeptides identified with neurological function (summarized in http://isyslab.info/NeuroPep/home.jsp or http://www.neuropeptides.nl, accessed on 29 November 2021) [80,81], and the potential to produce multiple neuropeptides from a single gene [82], highlight the importance of neuropeptide activity in neurological regulation.

The homeostasis of ion channel expression and the subsequent impact on neurotransmitter release play a crucial role in controlling cell membrane potential and intrinsic excitability. Neuropeptides can modulate ion channel expression in neurons by binding GPCRs on the neuronal membrane, which triggers a range of intracellular, second messenger signalling pathways. The result is the modulation of ion channel expression coupled to neuropeptide GPCRs [83], or the direct opening/closing of coupled channels [84], both of which determine neuronal excitability. The first example of this kind of neuropeptide was FMRFamide, which modulates Na^+^-selective ion channel opening in the snail cerebral neuron [85]. Since then, numerous studies have reported the direct and indirect regulation of acid-sensing ion channels by neuropeptides such as nocistatin [86,87], big dynorphin [88], and RFamide [89]. These neuropeptides are therefore of particular interest to researchers of stroke, as the activation of acid-sensing ion channels has been reported to facilitate acidosis and exacerbate neuronal death during ischaemic stroke [90]. Finally, neuropeptide S has been shown to regulate HCN channel activity in the amygdala of rats by inhibiting current flow through the channel, impacting the response to chronic pain [91].

In addition to their role in ion channel regulation, neuropeptides impact synaptic function and neurotransmitter release. The role of neuropeptides in synaptic regulation is exemplified by the retrograde control of synaptic transmission by dynorphin. Postsynaptic glutamatergic receptor activation depolarizes granule cell dendrites and triggers the exocytosis of dynorphin [92], which acts on presynaptic kappa opioid receptors to reduce the presynaptic probability of neurotransmitter release [93,94]. Additionally, recent studies propose a negative regulatory role of the amyloid-beta (Aβ) peptide in neuronal and synaptic function. Although the Aβ peptide is traditionally associated with the development of Alzheimer’s disease and not usually regarded as a neuropeptide, it is produced through the secretory pathway via the post-translational cleavage of the amyloid precursor protein [95] and co-secreted with classical neurotransmitters [96]. It can therefore be considered to fit with the traditional neuropeptide definition. Aβ is continuously produced and cleared in a non-diseased central nervous system throughout life. Independent studies showed that minute picomolar amounts of Aβ enhance memory stabilization [97,98]. The activity-dependent release of Aβ [99] and Aβ-induced reductions in synaptic and neuronal activity [100] also suggest the involvement of Aβ in the homeostatic regulation of neuronal activation. Only under conditions of disturbed homeostasis, such as the reduced clearance of Aβ relating to APOE4 expression, or the heightened production of Aβ, does the benign routine Aβ production trigger a series of pathological events driving neuronal demise [101]. 

Alongside neurotransmitter release, synaptic plasticity strongly influences behavioural outcomes towards environmental perception. The combined effects of various neuropeptide systems are central to the complex regulation of behaviour, and the tight control of excitatory/inhibitory activity is key to maintaining balance in the neural network. Interactions between brain-derived neurotrophic factor (BDNF) and OrphaninFQ provide an opportunity for sensory-dependent modulation of synaptic activity [102,103,104]. Neurohormones such as VIP homologs, oxytocin, and neuromedin modulate the activity level of synapses involved with associative learning [105], through the regulation of LTP and LTD processes within specific neuronal networks [106]. Neuropeptides may directly affect neuronal subpopulations to modulate network activity. For example, the gastrin-releasing peptide (GRP) modulates VIP and somatostatin interneuron activity to maintain excitatory and inhibitory (E/I) balances underlying the formation of memories in the neocortex [107]. Alternatively, NPY, corticotropin-releasing factor (CRF), and dynorphin regulate inhibitory GABAergic activity to maintain the balance of neural networks in the amygdala [108,109,110,111], and substance P prolongs the potentiation of glutamatergic NMDA receptors, enhancing neuronal excitability, in a number of brain regions including the SCN, striatum, and nucleus tractus solitaries [112]. The pivotal role of neuropeptides in regulating the balance of excitatory/inhibitory activity is further evidenced by their ability to attenuate the severity of seizures during large reductions in inhibitory activity, or epileptogenesis [113]. Further studies are required to examine the general applicability of neuropeptide modulation of synaptic function in different brain circuits.

### 2.3. Neuropeptides and Sensory Perception

As neuropeptides are involved in multiple components of neural homeostasis, they are integral to sensory perception and behavioural regulation. Nociception and pain perception are critical for the induction of responses against harmful or dangerous environmental stimuli. A major component of the pain response, and its integration with pain-related emotion and cognition, involves neuronal activity in the amygdala [114]. Correspondingly, a cocktail of neuropeptides are involved in the response to pain perception within the amygdala (reviewed by Neugebauer) [115]. In rodent models of pain, heightened pain responses and neuroinflammation occur concurrently with enhanced excitatory synaptic transmission between the nucleus parabrachialis, basolateral nuclei of the amygdala, and the central nuclei of the amygdala (CeA) [116,117]. The release of neuropeptides calcitonin gene-related peptide (CGRP) [118,119], substance P [120], and vasopressin [121,122,123] have been reported to upregulate the excitatory, glutamatergic drive to enhance pain-related plasticity in the CeA. In contrast, neuropeptide S-, somatostatin-, and oxytocin-expressing neurons inhibit these pain-enhancing factors and downregulate nociceptive tone [121,122,124,125]. CRF-producing neurons have been linked with both modulations to excitatory and inhibitory activity [126]. Further studies on the complex roles of opioid receptor ligands are required to fully understand their impact on synaptic plasticity and the subsequent pain response in the amygdala. Currently, β-endorphin has been suggested to exert analgesic effects via inhibiting substance P in the peripheral nervous system and GABA in the CNS, leading to excess release of dopamine [127], whilst enkephalins have been reported to promote analgesia by inhibiting substance P in the dorsal horn of the spinal cord [128]. 

Inflammatory processes and the response to pain are strongly correlated. It is unsurprising that neuropeptides involved in inflammation also play a central role in the pain response. Migraines and headaches emerge with the vasodilation of blood vessels in the brain following the release of CGRP and substance P by activated C and aδ sensory nerve fibers [129,130,131]. Interestingly, CGRP also exerts cardioprotective effects against hypertension in the peripheral system [132]. Adropin and 26RFa are alternate neuropeptides implicated in cardiovascular function and have consequently been suggested to act as mediators between neural and peripheral function [133], although the precise interactions between these neuropeptides and CGRP in the control of cardiac function are unknown. The prominent role of CGRP and substance P in the pain response has been reviewed by Carr and Frings [134], and their actions have also been associated with other peripheral sensory processes, including olfaction [135], gustation [136], retinal function [137], and the amplification of sound-evoked activity in cochlear nerves [138]. The role of CGRP in olfaction and gustation, in particular, may provide a direct link between neuronal sensory processing and energy dysfunction in metabolic disorders such as obesity [139].

The final multisensory integration of primary sensory stimuli creates a unique experience towards a world of independent perceptual entities. The extent to which multisensory defects occur has been associated with the severity of hallucinations in neuropsychiatric patients [140], however, the biomolecular basis of sensory stimulus integration into a single behavioural response has not been fully elucidated. In the *Drosophila* system, upon mechanosensory activation, neuropeptide F, an analogue of NPY produced by the primary sensory neuron DP-ilp7, activates relay A08n neurons to convey stimuli into a behavioural change [141]. A similar mechanism is adopted by the mammalian system; the distinct topological expression of urocortin 3 (UCN3) and its receptor corticotropin-releasing hormone receptor 2 (CRFR2) are critical for protecting auditory function under stress conditions [142]. A strong presence of CRFR2 within the subcortical auditory areas [143] reveals a potential mechanism for sensory cortex activation and auditory signal integration. This involves the activity-dependent release of neuropeptide signals and volume transmission towards neurons expressing the corresponding receptor, not necessarily situated near the source of the stimulus, involved in sensory processing and integration. Further studies are required to reveal the extent of specific neuropeptide modulation of sensory consolidation and integration within the mammalian nervous system.

### 2.4. Neuropeptides and the Systemic Inflammatory Response

Neuropeptides contribute directly towards the control of immune cell function. Microglia express receptors for various neuropeptides including NPY, opioids, VIP, and PACAP (reviewed by Carniglia et al. [144]), indicative of a potential neuropeptide role in controlling microglial function. Substance P, corticotropin-releasing hormone (CRH), and CGRP have been shown to activate mast cells and microglia, leading to the release of pro-inflammatory cytokines and chemokines including TNF-α, CCL2, IL-6, IL-1β, IL-33, and CXCL8 [144,145,146,147], which promote BBB permeation [148]. Interestingly, there is a convergence of the effects of small endogenous peptides such as VIP, substance P, and other cationic peptide drugs on mast cell activation via the Mas-related gene X2 receptor (MrgprX2) pathway [149], suggesting the presence of common pro-inflammatory pathways evoked by neuropeptides. Inflammatory processes have been reported to enhance tissue damage and accelerate the pathology of numerous neurological disorders including AD [150,151,152,153], stroke [154,155], Parkinson’s disease (PD) [156,157], amyotrophic lateral sclerosis (ALS) [158], Huntington’s disease (HD) [159], and neuropathic pain [160], as well as metabolic disorders such as obesity [161,162] and normal aging [163], all of which have been reported to display altered levels or function of neuropeptides. VIP has been heavily implicated in the regulation of the inflammatory response in the periphery (a comprehensive review can be found by Hooper and Kong (2015) [164]). For example, in mice VIP has recently been shown to modulate the production of IL-22 from lymphoid cells in the gastrointestinal tract, which is important in immune homeostasis of the gut [165]. VIP expression appeared dependent on cyclic food intake, whilst the upregulation of circadian clock genes also correlated with IL-22 production, highlighting the contribution of circadian rhythms and associated neuropeptides to the inflammatory response. Consequently, dysfunction of neuropeptides may have multiple effects on disease pathology, which are likely to be linked; their activity leads to the exacerbation of neuronal death and disruption of neuronal homeostatic mechanisms, but also contributes towards systemic inflammation, further aggravating these processes. Harnessing or upregulating the effects of anti-inflammatory neuropeptides such as VIP, NPY, and somatostatin [166,167,168,169,170], or the inhibition of mechanisms commonly activated, may be of therapeutic benefit.

The systemic inflammatory response occurs when local control of inflammatory processes is lost, resulting in an excessive, widespread release of inflammatory mediators and subsequent inflammation [171]. The process is mediated by leukocytes in the periphery [172,173,174,175], microglial cells in the brain [176], and mast cells associated with both the brain and peripheral system [145], resulting in the widespread recruitment of inflammatory mediators in the CNS and peripheral tissue. Neuronal damage as a result of the systemic inflammatory response occurs due to increased circulation of peripheral pro-inflammatory cytokines and chemokines. These disrupt the BBB by inducing tight junction modifications, endothelial damage, astrocyte changes, and degradation of the glycocalyx and glia limitans, as reviewed by Varatharaj and Galea [174], and hence promoting proinflammatory processes in the brain [151]. Another way in which these inflammatory mediators cause neuronal damage is via the degradation of tryptophan into harmful intermediate metabolites, which can directly cause dysfunction of neurotransmitter receptors and modulate redox processes and the activity of immune cells [152]. Interestingly, the upregulation of neuropeptides β-endorphin, orexin, and oxytocin has been shown in rats during the systemic inflammatory response to acute pancreatitis [177], and this process appears independent of and earlier than the release of cytokines from microglia, indicative of a role of neuropeptides in the earliest stages of inflammation. Enkephalins are also involved in various components of the inflammatory response, including neutrophil adherence to endothelial cells [178], T-cell migration, and IL-6 secretion [179]. As a counter response, some neuropeptides, such as VIP, NPY, α-Melanocyte-stimulating-hormone (α-MSH), somatostatin, and kyotorphin-amide [180,181,182,183,184,185,186] are anti-inflammatory and inhibit microglial or leukocyte activation.

## 3. Interrelation between Neuropeptide Signalling, Metabolic Dysregulation, and Neurological Dysfunction

Due to their prominent role in the control of neural networks underlying food intake and motivated behaviours, dysfunction of certain neuropeptides has been strongly linked to the development of obesity, a systemic disorder which is increasingly being viewed also as a disorder of the central nervous system. For example, changes in the density of the µ-opioid receptor in areas of the brain including the ventral striatum and thalamus have been shown in obese patients [187]. This is indicative of altered function of endorphins and endomorphins acting on these receptors and a likely link between circuitry underlying reward and the release of insulin and leptin to control food intake. Indeed, opioid peptides have been reported to regulate insulin, leptin, and glucose homeostasis [188,189]. Interestingly, recent studies have suggested that metabolic dysfunction observed in obesity is associated with cognitive decline symptomatic of Alzheimer’s disease. The two may be linked through circuits controlled by insulin and leptin signalling mechanisms; both have been reported to play important roles in neuronal homeostatic mechanisms, such as synaptic plasticity underlying learning and memory processes [190]. Neuropeptides 26RFa and adropin have been implicated in dysfunctions of energy homeostasis, with increased levels of 26RFa observed in obese, compared to healthy, patients, and modulations in adropin levels seemingly dependent on diet. Expressed in neuronal populations in the hypothalamus, these two neuropeptides have been suggested to play peripheral roles in metabolic homeostasis and cardiovascular function, and so it is likely that they enable cross-talk between the brain and peripheral tissues such as the gut and heart [133]. The regulation of insulin and glucose homeostasis by 26RFa and adropin has been heavily reported [191,192,193,194], and so abnormal control of insulin release by neuropeptides may play a central role in the dysfunction of metabolic regulation and energy homeostasis in obesity, as well as in the onset of cognitive decline observed in AD patients. In fact, disruptions to cognitive function as a result of aging have already been shown to be reversed following intraperitoneal administration of adropin peptide in mice, indicative of a potentially prominent role of the neuropeptide in the maintenance of neural homeostasis during aging [195]. Similar has recently been shown of neuropeptide 26RFa; microinjections of the neuropeptide into the hypothalamus of rats improved short-term memory and performance on the Morris water maze task [196]. 

Additionally, neurological dysfunction of the liver-expressed antimicrobial peptide 2 (LEAP-2) and its effects on the ‘hunger hormone’ ghrelin have also been highlighted as possible contributors towards the development of obesity. Actions of ghrelin on its growth hormone secretagogue receptor (GHS-R) are known to stimulate food intake, but have also been recently reported to promote spatial learning and memory in the hippocampus [197]. LEAP-2, produced in the liver and small intestine, antagonizes GHS receptors, therefore reducing ghrelin levels and subsequently suppressing the want to consume food [198]. The aetiology of obesity remains complex and incompletely understood, although studies have reported reduced levels of ghrelin and increased LEAP-2 in obese mouse models and human patients [198,199,200,201]. Due to the role of ghrelin in cognition, its increased inhibition by upregulated LEAP-2 levels may be a crucial link between obesity and cognitive dysfunction, implicating a potentially critical role of the LEAP-2 peptide in communication between the brain and gut in both metabolic and cognitive control.

Instead of being viewed as classical hormones which are able to bind receptors in the brain, insulin, ghrelin, and leptin are increasingly being seen as having roles in whole-body homeostatic mechanisms [202], as well as neuropeptide-like roles in neuronal homeostasis. For example, a recent study in Drosophila has supposed that upd2, of which leptin is the mammalian ortholog, and insulin exert opposing roles on fat-sensing neural networks in order to modulate synapse number and neuronal negative tone [203]. The control of peptide-like hormones such as insulin, leptin, and ghrelin by neuropeptides including 26NFa and adropin, as well as LEAP-2, appears to be crucial in the regulation of energy homeostasis, metabolism, and cognitive function. Consequently, increased understanding of the circuitry and signalling mechanisms underlying such processes, and how they interact and are linked, has the potential to translate into improved treatments for both neurological and metabolic diseases. 

Neuropeptide activity is essential for maintaining neuronal homeostasis, controlling cellular signalling mechanisms, and subsequently regulating a wide range of biological functions, and so it is clear that its dysfunction is likely to be observed in, and possibly a contributing factor towards, a number of neurological diseases. For example, one review by Manuel et al. [204] indicates that, in the cerebral cortex of Alzheimer’s disease patients and/or rodent models, levels of neuropeptides such as galanin, dynorphin, and hemorphin are increased. Galanin has been reported to play a role in learning and memory processes via modulations of hippocampal cholinergic pathways [205], and so it could be hypothesized that alterations in the levels and function of the neuropeptide contribute towards memory loss in AD, arguably the disease’s most defining symptom. Opioid peptides such as dynorphin and hemorphin likely contribute to imbalances in excitatory/inhibitory (E/I) function and subsequent cognitive deficits in AD, as they have been supposed to disrupt the activity of a number of neurotransmitters over the course of the disease, including glutamate, GABA, acetylcholine, and noradrenaline [204]. Conversely, levels of CRF, cholecystokinin, and somatostatin have been reported to be reduced in the cerebral cortex of AD patients and rodent models, and it is therefore plausible that their reductions also contribute towards disease pathology; all three have previously been associated with cognitive function, including circuitry underlying learning and memory [206,207,208]. Interestingly, levels of NPY and substance P were reported to be reduced in the cortex of AD patients, but increased in that of certain AD mouse models [204]. This may reflect the complex role of these neuropeptides in a range of cellular signalling pathways and suggests their actions and modulation in AD may be multiplex in terms of influence on disease pathology (however, it must be considered that mechanisms which drive certain aspects of pathology may just simply differ between species). 

Neuropeptide Y dysregulation in particular has been linked to amyloid beta (Aβ) pathology. The oligomeric form of Aβ is viewed as a potential contributor of cognitive decline observed in patients, due to its ability to induce neuronal death, disrupt synaptic plasticity, and subsequently advance brain atrophy [209]. Aβ has recently been proposed to adversely impact NPY function via the modulation of voltage-gated calcium channel activity on NPY-expressing neurons [210]. However, NPY has been suggested to attenuate the toxic effects of Aβ accumulation [211], and so further research into interactions between the two molecules will be beneficial; the therapeutic potential of NPY has already been discussed in a number of review papers [186,212,213]. Neuropeptide Y dysfunction has in fact been reported in several other neurological disorders, including PD, HD, Machado-Joseph disease (the potential role of NPY in all three is discussed by Duarte-Neves et al. [186] and Li et al. [211]), and ALS [214], reflective of its neuroprotective role in neural networks. Due to their diverse range of functions, it is common for modulation of the same neuropeptide to be associated with more than one neurological disorder. Another example is that of prolactin. When functioning normally, prolactin contributes towards the control of numerous biological circuits, including those underlying anxiety, neurogenesis, food intake, maternal behaviour, and pain [215]. However, alterations in its activity have been associated with pathology in both AD and PD (reviewed by Nguyen et al. [216]), the two most common neurodegenerative diseases. 

Alterations in neuropeptide function have also been associated with pathology in stroke and epilepsy. As previously mentioned, the regulation of acid-sensing ion channels (ASICs) is altered during ischaemic stroke, contributing to neuronal death, and has been shown to depend on neuropeptide function (nocistatin, big dynorphin, and RFamide). Additionally, the upregulation of substance P, bradykinin, and neurotensin have all recently been reported to worsen stroke pathology by increasing BBB permeability [217], although their control and neutralization via actions of neurolysin appears to reduce resultant oedema [218]. Finally, studies have reported that polymorphisms in the NPY gene promoter may increase risk of ischaemic stroke [219,220,221], and upregulation of the neuropeptide has been suggested to contribute towards mechanisms underlying haemorrhagic stroke onset [222], whilst lower levels have been linked with post-ischaemic stroke epilepsy [223]. Epilepsy occurs as a result of imbalances in excitatory and inhibitory neuronal activity, and as a result of their role in maintaining excitatory/inhibitory balances in the neural network, the dysfunction of numerous neuropeptides has been linked with the onset of epileptogenesis. For example, levels of neuropeptides which tend to reduce excitatory activity, such as NPY, dynorphin, and galanin become reduced, whereas those which increase excitatory activity, such as substance P, become upregulated [224]. This results in abnormally high excitation, and hence seizure onset. Consequently, targeting neuropeptide function in the treatment of seizures is promising, but, currently, more research into the specific mechanisms of neuropeptide action during epileptogenesis and resulting impacts on glutamatergic and GABAergic activity in particular is required.

## 4. Potential of Neuropeptides in Resolving Outstanding Questions in Neurological Research

The role of neuropeptides in mechanisms underlying numerous brain functions is likely to be as necessary as that of neurotransmitters, yet neurotransmitter function tends to receive more attention in neurological research. A large number of pharmaceutical drugs are small molecules [225] which, when harnessed for neurological use, target neurotransmitter receptors and modulate synaptic transmission. However, due to the complexity of symptoms and pathogeneses involved in neurological disease, the effectiveness of small molecule treatment is often inconsistent between patients. For example, not all patients display clinical effects with treatment using the FDA-approved Alzheimer’s disease drugs targeting cholinergic or glutamatergic neurotransmitter systems, and there is little basis for drug choice between patients [226]. Treatment options are further limited by the lack of drug specificity, which can often lead to undesirable off-target effects. Peptides generally have a higher specificity for their targets due to their coevolution inside the cell with their relevant receptors, allowing optimized specificity for precise interactions [227,228,229]. Additionally, their ability to exert longer-lasting effects due to a lack of reuptake mechanisms allows neuropeptides prolonged control of neuronal circuits from relatively large distances, which is difficult to achieve via the faster actions and reuptake of neurotransmitters (although it can be in specific situations, such as during the release of dopamine from axonal varicosities [37]). The likely sustained drug effects in target sites are opposed to the frequent dosing required for more rapidly metabolized small molecule drugs. Upon inactivation, neuropeptides can produce alternate bioactive peptides for alternate functions. 

Further research into neuropeptide function can address outstanding neurological questions. How do neuropeptides interact with each other, with neurotransmitters, and other proteins to control neuronal function? Interestingly, the co-storage of CGRP, substance P, and BDNF inside single large granular vesicles has been reported [230], although it is undetermined whether differential stimulation of vesicle release can lead to the release of lone neuropeptides within a co-stored vesicle as opposed to releasing all of its contents. It would be interesting to determine how such possible mechanisms may be relevant to differential postsynaptic neuronal function. Additionally, there remains an incomplete understanding of the crosstalk occurring between multiple neuropeptides that underlies behavioural processes [231]. However, it appears that the co-storage and simultaneous release of various neuropeptides presents a way in which neuropeptides can act together in a precise manner to exert actions on post-synaptic neurons. The release of neuropeptides, and whether this is independent or alongside various combinations of other neuropeptides or neurotransmitters, likely impacts the resulting response of postsynaptic neurons. This serves to increase the diverse functions of neuropeptides. Furthermore, tissue-specific cleavage of pro-peptides can potentially generate distinct populations of the final mature peptogenic modulator and increase the eventual diversity and effects of a single pro-peptide [232]. 

Due to their high level of diversity in terms of actions on a wide range of receptors, and the involvement of singular neuropeptides in multiple cellular pathways, care must be taken when modifying peptide function. The modulation of one neuropeptide may also become problematic if this peptide is co-released alongside other peptides in which changes to levels and function may be undesirable. Additionally, the larger size of neuropeptides compared to neurotransmitters may make synthesis more difficult. However, the large diversity of neuropeptides, obtained via their differential processing from pro-peptides, ability to co-exist in and be co-released from large granular vesicles, and capacity to exert mechanisms of action across larger spatiotemporal scales with higher specificity than neurotransmitters, reflects their unique functioning in both central and peripheral systems. For this reason, research into the therapeutic potential of neuropeptides in a range of disorders will advance knowledge beyond just that of neurotransmitter modulation of neuronal circuits.

## 5. Synthetic Approach, Strategies, and Prospects for the Development of Peptide-Based Neurotherapeutics

A significant number of neuropeptides identified are relatively large in size. However, effective peptide synthesis with more than 30 amino acids is difficult with traditional chemical methods such as solid-phase peptide synthesis (SPPS) due to the aggregation of incomplete products and accumulation of byproducts that interfere with the chemical reaction [233]. Instead, recombinant techniques through the artificial expression of target peptides in bacterial systems have been successfully adapted to produce larger-sized peptides in significant amounts [234,235]. In 1982, the FDA approved the use of recombinant human insulin, with a total of 51 amino acid residues, for the treatment of diabetes melitus [236]. Further advancements in genetic engineering allowed single amino acid-level manipulation to optimize peptide properties for therapeutic use [237,238]. These advances were applied to the development of fast-acting insulin analogues [18]. The FDA approval for recombinant salmon calcitonin for treating hypercalcemia and postmenopausal osteoporosis in 2005 [29] and the parathyroid hormone (PTH) for osteoporosis in 2002 [27] are two success stories of the recombinant technology in the production of peptide therapeutics. Furthermore, new enzymatic ligation methods allow the production of complex neuropeptides at a reduced cost [31]. Exenatide, a synthetic version of exendin-4 (E4), has been successfully synthesized on a gram scale through the enzymatic ligation of synthetic peptide fragments [239].

The poor chemical and physical stability, short circulating half-life, and low oral bioavailability of natural peptides [240] initially restricted the applicability of peptide therapeutics in medicine. A huge focus has been placed on the enhancement of metabolic stability of peptide therapeutics. To increase circulation half-life, various modifications are added to cleavage sites. These modifications include N-terminal acetylation [241], N-methylation [242,243], incorporation of D-amino acids or non-natural amino acids [244], and uncleavable bond mimetics such as thioamides and peptoids [245,246]. Alternatively, N- to C-terminal cyclization [247] and disulfide bond mimetics [248] have been used to modulate stability and bioavailability. Besides the in vivo stability, most peptide therapeutics suffer from short blood circulation time due to rapid renal clearance [249]. A promising approach to combat this problem is to piggyback peptides onto serum proteins with long plasma half-lives, by conjugating ligands that bind to serum proteins [250]. Insulin determir, insulin degludec, liraglutide, and semaglutide are alternative forms of insulin conjugated to albumin-binding fatty acids to improve insulin pharmacokinetics, which have been approved for clinical use [19,20,34]. Recently, a serum albumin-binding short peptide has been designed and combined with a fatty acid to further increase the binding affinity of therapeutic peptides to serum albumin and hence increase the half-life in serum [251]. Another promising approach is to selectively conjugate peptides to serum albumin or immunoglobulin with a longer circulation time. Dulaglutide consists of two identical GLP-1 analogues covalently linked to a modified Fc fragment of human IgG4. Albiglutide consists of a GLP-1 dimer fused to recombinant human albumin. Both the protein-fused GLP-1 analogues showed extended half-lives allowing less frequent dosing [252]. 

The majority of peptide or peptide-based drugs are administered by injection, which can lead to unwanted side effects, such as pain, infection, or serious allergy [253]. Alternative routes of peptide delivery have been developed and tested. Throughout the years, various new methods have been employed to increase the ease of the delivery of insulin, such as the use of an implantable device [21], needle-free jet injection [22], and microneedle-based transdermal delivery [23,24]. Pulmonary delivery of inhalable insulin has been considered as an attractive delivery method, however, it has raised safety concerns with side effects resulting in commercial failures [25]. Oral delivery is the most favorable method due to its convenience. However, peptides are rapidly degraded by enzymes in the gastrointestinal tract and do not permeate the intestinal epithelium easily [254]. An approach to improve oral bioavailability is the co-formulation with intestinal permeation enhancers. In 2019, the FDA approved the first oral GLP-1 receptor agonist, Rybelsus, for the treatment of type 2 diabetes mellitus. It is a semaglutide tablet co-formulated with a small fatty acid derivative as a permeation enhancer [33]. In 2020, the FDA also approved an oral octreotide capsule that is encapsulated with proprietary excipients (transient permeability enhancer) for the treatment of acromegaly [33]. An oral insulin (ORMD-0801) with a permeation enhancer is currently in phase III to treat type 2 diabetes [255].

The selective permeability of the BBB is the biggest obstacle to the efficient delivery of drugs into the brain [256,257,258]. Self-assembling peptides [259], shuttle peptides [260], and peptide nanoparticles [261] have recently succeeded in transpassing the BBB and reaching the brain parenchyma, showing the potential of peptide therapeutics in neurological treatment. For example, apolipoprotein B (ApoB) is an effective BBB shuttle peptide, and the administration of ApoB-fused NPY successfully increased NPY activities in the brain of an Alzheimer’s disease mouse model, which reversed neurodegenerative pathology [262]. Polymeric nanoparticles have been applied to the delivery of oxytocin into the brain [263]. Poly(lactic-co-glycolic acid) (PLGA) or bovine serum albumin (BSA) are used as the base material of these nanoparticles, and transferrin or rabies virus glycoprotein (RVG) are conjugated to the base material to specifically deliver oxytocin into the brain. Intranasal administration bypasses the BBB to allow the direct delivery of neuropeptides into the brain. This method enhances bioavailability and reduces side effects from the treatment [264]. Since the first clinical trial of intranasal administration of insulin was reported in 2002 [265], subsequent studies have been conducted to treat AD patients [265,266]. Intranasal administration of oxytocin has also been clinically studied to treat schizophrenia [267], post-traumatic stress [268], and autism spectrum disorder [269]. Currently however, oxytocin analogues are only FDA-approved for the induction of uterine contractions and prevention of excess bleeding during childbirth [16].

Ancient peptides phylogenetically related to known neuropeptides, which contain natural structural elements capable of bypassing the BBB, provide valuable insights for drug development. They are likely to play fundamental roles in neuronal energy homeostasis and may provide further insights into neuropeptide actions in the CNS [248]. For example, the teneurin C-terminal associated peptide (TCAP), which can naturally cross the BBB, is associated with the evolutionary derivation of CRF and related neuropeptides [270]. Combining increased knowledge of neuropeptide actions with computational modelling of candidate peptides may reduce the current limitations associated with the development of peptide therapeutics [271]. The computational modelling of peptide macrocycles amalgamates the benefits of small molecules (higher permeability) and larger molecules (increased interaction surface area) while improving the understanding of their possible multiplex actions. This could lead to both increased target affinity and reduced drug development time [272]. The recent development of computer algorithms that can generate and screen novel macrocyclic molecules from small peptides could lead to significant advancements in the effectiveness of peptide therapeutics by enhancing understanding of how the structure of macrocyclic peptides relates to their activity [273]. 

Co-targeting neuropeptides with adjunct therapies may enhance treatment options given the frequent pleiotropic roles of neuropeptides in a myriad of signalling cascades (Table 2). The addition of molecules targeting complementary pathways to GLP-1, such as cholecystokinin, glucose-dependent insulinotropic peptide, glucocorticoids, and oestrogen, are proposed to enhance antidiabetic actions of GLP-1 agonists and reduce the gastrointestinal side effects commonly observed with agonist application [274,275]. GLP-1 agonists are also used to treat opioid-use disorder (OUD), although their clinical effectiveness is limited by such side effects. A recent study in rats suggested that the co-targeting of GLP-1 receptors and neuropeptide Y2 receptors using a dual agonist peptide reduced opioid seeking and attenuated adverse effects and may be a more suitable way of treating OUD in human patients [276].

Due to their prominent role in the pain response and inflammatory processes, opioid peptides have high therapeutic potential, although such treatments are tightly regulated due to the risk of addiction. Nonetheless, the use of non-addictive opioid analogues may open new avenues for opioid-related therapeutics. A recent study showed that the application of a dermorphin and substance P fragment analogue, when co-applied with biphalin (an enkephalin analogue), accelerated wound healing in diabetic rats [277] while biphalin alone reduced arterial pressure in rats [278]. Aside from actions on classical opioid receptors mu (µ), delta (δ), and kappa (κ), enkephalins and dynorphins bind various other receptors, including the Mas-related G protein-coupled receptors, bradykinin receptors, and NMDA-Rs. For example, the analgesic effects of methadone depend not only on the activation of classical mu opioid receptors but also simultaneous antagonism of NMDA-Rs [279]. Reviewed in detail by Palmer et al. [280], the consideration of opioid actions on these atypical receptors may enable the enhancement and optimization of opioid peptide therapeutics. Therefore, hybrid and analogue opioid peptide approaches and targeting of non-classical sites of opioid action show promise in enhancing the efficiency of therapeutics associated with pain and the inflammatory response.

## 6. Conclusions

**Figure 1:**
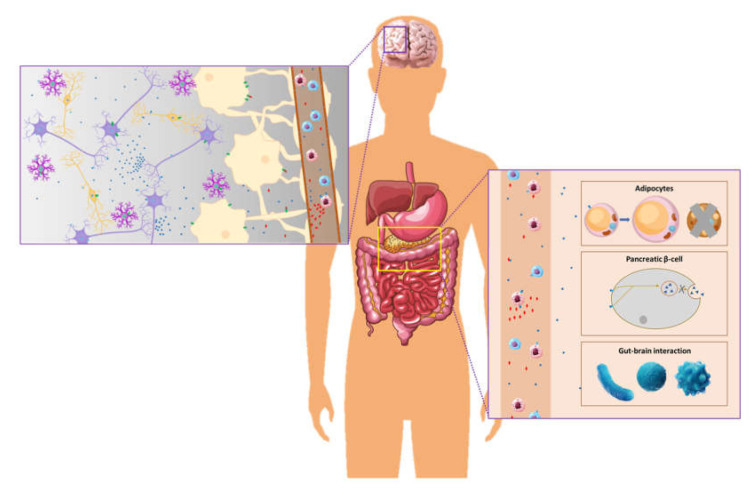
Widespread roles of neuropeptides in human physiology. Neuropeptides produced in the nervous system can modulate local neuron or glia function, through autocrine or paracrine effects. Through volume transmission, these neuropeptides find their way into the peripheral organs to modulate the function of peripheral organ systems (endocrine effects).

As a result of their wide range of roles in both peripheral and neurological function (Figure 1), neuropeptides have high therapeutic potential for many disorders. At an early stage of brain development, neuropeptides regulate synapse formation and neuronal proliferation and differentiation. During adult life, they have been shown to modulate ion channel activity, neurotransmitter release, and synaptic plasticity, all of which contribute to maintaining the E/I balance within the brain. Additionally, they have been strongly suggested to control immune cell function during the systemic inflammatory response and possibly act as essential mediators of communication between the CNS and PNS. Their therapeutic potential therefore spans from the treatment of neurodevelopmental disorders such as autistic spectrum disorder to neuroinflammation associated with stroke and to a wide range of neurological diseases associated with changes to the E/I balance such as epilepsy and neurodegenerative disorders. Importantly, due to the fact that a number of peptides also play a role in peripheral cell function, they may also be harnessed as therapeutic agents for the treatment of metabolic disorders. Targeting peptides which are expressed in both the CNS and periphery may be a beneficial way to address peripheral and cognitive deficits arising as a result of metabolic diseases such as obesity which, in recent times, are being progressively linked with the appearance of cognitive symptoms. 

The effectiveness of peptide therapeutics has been increasingly recognized with recent optimizations such as single amino acid modifications, enhancing delivery and pharmacokinetics of current FDA-approved treatments. Nonetheless, neuropeptide modulation of the nervous system remains a relatively new concept. It is not surprising that current peptide therapeutics for neurological disorders target alternative, well-characterized systems and mechanisms, the majority of which focus on peripheral symptoms, including those in the treatment of diabetes mellitus with insulin or GLP-1. Further understanding of new therapeutics will be required to improve treatment options for neurological disorders. Currently, increased research into alternative components of neuropeptide function such as the modulation of phylogenetically ancient neuropeptides, the development or use of macrocyclic peptides, the benefits of adjunct therapies targeting multiple neuropeptide pathways, and the production of synthetic opioid peptides and their possible actions on non-classical receptors have been actively underexplored compared to well-validated systems. Combining research into these areas with novel machine learning and computational modelling techniques is likely to further enhance the therapeutic potential of neuropeptides in neurological and peripheral diseases.

## Figures and Tables

**Table 1:** Representative FDA-approved peptide-based drugs.

Peptide	Trade Name	Target Condition	Modifications to the Original Structure	Year of Approval	Remarks
Vasopressin	Desmopressin	Diabetes insipidus [13], nocturia [14]	No modifications	Diabetes insipidus in 1978, nocturia in 2017.	Purified posterior pituitary extract was used before the synthetic production of vasopressin [15].
Oxytocin	Pitocin	Obstetrics, to induce labor and prevent postpartum bleeding [16]	No modifications	1980	First peptide hormone synthesized in the lab [17].
Insulin [10]	Insulin lispro, insulin aspart, insulin glulisine (rapid-acting), insulin glargine, insulin detemir, insulin degludec (long-acting)	Type I and II diabetes mellitus	Single amino acid modifications increase speed of release [18]. Insulin determir and degludec conjugated with fatty acids to enhance albumin binding, improving pharmacokinetics [19,20].	First analogue (insulin lispro) approved in 1982.	Alternative delivery methods attempted: implantable device, needle-free jet injection, transdermal delivery, microneedle-based delivery, pulmonary delivery [21,22,23,24,25]. There has been a shift in reliance, from synthetic human insulin towards insulin analogues. In 2010, 91.5% and 14.8% of type II diabetes patients use analogues and human synthetic peptides respectively, compared to 18.6% and 96.4% in 2000 [26].
Parathyroid hormone [27,28]	Teriparatide	Osteoporosis	34 amino acids in the N terminus is used.	2002	Designed using recombinant technology.
Calcitonin [29]	Fortical (recombinant salmon calcitonin)	Hypercalcaemia, postmenopausal osteoporosis	No modifications	2005	Designed using recombinant technology. Restricted use due to increased cancer risk [30].
Exendin-4 [31]	Exenatide	Type II diabetes mellitus	Synthetic version of exendin-4.	2005	Glucagon-like peptide 1 (GLP-1) receptor agonist. Synthesized on gram scale via enzymatic ligation of synthetic peptide fragments.
Adrenocortico-tropic hormone (ACTH)	Achtar gel	Infantile spasms [32]	No modifications	2010	ACTH initially isolated and introduced to treat endocrine disorders in the 1950s [10].
GLP-1	Liraglutide, semaglutide (Rybelsus)	Type II diabetes mellitus, obesity	Conjugated with fatty acids in order to enhance binding to albumin, improving pharmacokinetics [33,34].	Liraglutide in 2014, semaglutide in 2019.	Glucagon-like peptide-1 analogue [34].
Somatostatin [35]	Octreotide	Acromegaly, diarrhea associated with metastatic carcinoid tumors and VIP-secreting tumors	Encapsulated with proprietary excipients (transient permeability enhancer).	2020	-
Difelikefalin [36]	Korsuva	Moderate to severe itching associated with chronic kidney disease	No modifications	2021	Κ-opioid receptor agonist.

**Table 2:** Potential neuropeptides and proteinaceous molecules that can be used in neurotherapeutics.

Peptide/s	Possible Targets and Mechanisms of Action	Systems Affected	Potential Application
NPY	Neuroprotective [211], regulation of immune cell function [145].	Widely expressed in the peripheral system and the central nervous system, including the hippocampus, hypothalamus, amygdala, striatum.	Wide range of neurological disorders, including AD, PD, HD, Machado-Joseph disease [186,211,212], ALS [214], as well as associated inflammatory processes [168,169]. Potential anticonvulsant effects against seizures [281].
Oxytocin	Modulation of LTP and LTD of synapses during early development [66,105,282]. Suggested role in early stages of the systemic inflammatory response [177].	Predominant synthesis and expression in the hypothalamus. Expressed in lower densities brain wide.	Schizophrenia [267], post-traumatic stress disorder [268], and ASD [61,65,269].
Adropin and neuropeptide 26RFa	Regulate insulin and glucose homeostasis and cardiovascular function in the periphery [133,191,192,193,194]. Reported in the maintenance of neuronal homeostasis during aging [195,196].	Peripheral tissues associated with metabolic control and energy homeostasis. Hypothalamus, possibly hippocampus.	Metabolic dysfunction associated with obesity. Cognitive dysfunction as a result of aging.
LEAP-2 and ghrelin	Balance of ghrelin antagonism by LEAP-2 not only controls food intake [198,199,200], but has also been suggested to regulate spatial learning and memory [195,196].	Peripheral tissues associated with metabolic control. Hippocampus.	Obesity, learning, and memory problems associated with AD.
Prolactin	Reported roles in neurogenesis and neuronal stem cell proliferation. Expressed on microglia and astrocytes with suggestive roles in inflammatory response [215].	Hypothalamus, hippocampus, cortex.	AD, PD [216].
PACAP	Regulates synaptic plasticity via the modulation of glutamatergic transmission during development [68] and adulthood [283]. Reported role in immune response receptors expressed on microglia [144].	Widely expressed in the brain, including in the hippocampus and hypothalamus.	AD and PD [284], HD [285], Fragile X syndrome [283].
TLQP-62	Regulates developmental synaptic plasticity [69], neuroinflammatory and oxidative responses [286].	Hippocampus.	Neuropsychiatric disorders.
Neurolysin	Regulates activity of other neuropeptides, control of inflammation and excitotoxicity during ischaemic stroke [218].	Brain-wide effects.	Ischaemic stroke.
Nocistatin, big dynorphin and RFamide	Activate acid-sensing ion channels during stroke, facilitating acidosis and exacerbating neuronal death [86,87,88,89].	Brain-wide effects.	Inhibiting actions of these neuropeptides has the potential to reduce ASIC activation during ischaemic stroke, which may reduce subsequent pathological and inflammatory effects [90].
CGRP and substance P	Regulate inflammatory processes [144,145,146,147]. Enhance neuronal excitability underlying the response to pain [118,119,131,134].	Brain-wide effects. Actions in the nucleus parabrachialis, BLA, and CeA are particularly important in regulation of the pain response.	Inhibiting actions of CGRP/SP may reduce pathological effects of inflammatory disorders [287] and the pain response, e.g., as a result of migraines [288].
VIP and somatostatin [166,167,169]	Anti-inflammatory mediators of immune cell function.	Many peripheral locations, including GI tract, heart, kidneys, thyroid gland. Brain areas include hypothalamus, pituitary gland.	Neurological and peripheral disorders with associated inflammatory processes or autoimmunity.
Galanin	A primarily inhibitory role, possibly via the activation of serotonergic pathways [289].	Widely expressed in the peripheral and central nervous system, including in the medial temporal lobe.	Epileptic seizures [224].
Biphalin	An enkephalin analogue. Enkephalins have been implicated in pain responses [127,128], stress [290], and the inflammatory response [178,179].	Enkephalins are highly expressed in the limbic system of the CNS, peripheral organs such as the skin, liver, and lungs, and the adrenal medulla [290].	May accelerate immune system activation, a reduction of which has been associated with diabetes [277,291]. Hypertension [278].

## Data Availability

Not applicable.
